# The potential of biochar in improving drainage, aeration and maize yields in heavy clay soils

**DOI:** 10.1371/journal.pone.0196794

**Published:** 2018-05-11

**Authors:** Alfred Obia, Jan Mulder, Sarah Elizabeth Hale, Neneng Laela Nurida, Gerard Cornelissen

**Affiliations:** 1 Department of Environmental Engineering, Norwegian Geotechnical Institute (NGI), Oslo, Norway; 2 Faculty of Environmental Sciences and Natural Resource Management (MINA), Norwegian University of Life Sciences (NMBU), Aas, Norway; 3 Indonesian Center for Agricultural Land Resources Research and Development (ICALRRD), Bogor, Indonesia; RMIT University, AUSTRALIA

## Abstract

Heavy clay soils are globally widespread but their poor drainage and poor aeration limit their use for agriculture. This study was designed to test the effect of the amendment of biochar (BC) from woody shrubs on drainage/saturated hydraulic conductivity (K_sat_), soil aeration/air capacity, available water capacity and biomass and grain yields of maize. In a field experiment, BC from *Gliricidia sepium* was applied in planting basins or rip lines at 2.5% and 5% w/w in addition to a control without BC. The maize biomass and grain yields were higher in BC treated plots compared to control (p<0.05) during the 2012 and 2013 seasons. There was no significant difference in the yields between 2.5% and 5% BC treatments (e.g. grain yield were 6.6 and 8.1 t ha^-1^ in 2012 and 9.3 and 10.3 t ha^-1^ in 2013 compared to control with 4.2 and 6.7 t ha^-1^ in 2012 and 2013, respectively). Soil from the same field site was also mixed with a similar woody shrub BC from *Eupatorium adenophorum* in the laboratory at rates of 2.5%, 5% and 10% BC w/w and a control without BC. The mixtures were then incubated and subjected to two wet-dry cycles for two weeks. Core samples were taken from the incubated soil and tested for bulk density, K_sat_ and pF measurements. Total porosity and moisture at field capacity and wilting point were 72.3%, 43.7% and 23.7%, respectively, and not affected by BC amendment (p>0.05). In contrast, bulk density decreased linearly by 0.011±0.002 g cm^-3^ per percent BC added (p<0.001). K_sat_ and air capacity of the soil were 288 cm day^-1^ and 30.9%, respectively falling within the generally accepted optimal range. Both K_sat_ and air capacity followed a significant quadratic relation (p<0.05) upon BC addition, decreasing at low BC doses, reaching a minimum at 3–5% BC and increasing at higher doses. Results allowed a partial attribution of the yield increases to changes in soil physical properties such as changes in bulk density and not clearly to K_sat_ and air capacity.

## Introduction

Heavy clay soils that exhibit swelling and shrinking properties, referred to as Vertisols, are globally widespread. Amidst a limitation in soil data which varies from country to country, the current global distribution of Vertisols is estimated at approximately 300–350 million hectares [1,2]. Poor drainage and hence poor soil aeration are some of the main challenges when utilizing Vertisols for farming, yet these soils are often chemically fertile due to their high inherent nutrient holding capacity [2,3].

Organic amendments have been used to address some of these challenges. An example is the use of manures to improve structure and drainage of Vertisols [4,5]. Biochar (BC), a pyrolysis product of organic materials, could also be an amendment that can improve Vertisol utilization. In fact BC has been reported to increase total porosity of clayey soil [6], which may aid drainage. Similarly, in coarser soils, an increase in porosity has been reported e.g. [7–9].

One common way of measuring the drainage potential of a soil is by measuring its saturated hydraulic conductivity (K_sat_). Biochar prepared from wood has been reported to increase K_sat_ of clayey soil especially when added at high doses [10]. Ouyang et al [11] and Ouyang & Zhang [12] also observed an increase in K_sat_ by up to about 50% upon addition of woodchip and dairy manure BC to clayey soils in incubation experiments. Such increase in K_sat_ indicates that BC may increase drainage of Vertisols. In contrast, Asai et al [13] and Castellini et al [14] observed no effect of wood BC on K_sat_ of clayey soils. For coarser soils, similar conflicting results on the effect of BC on K_sat_ have been reported. For example, Barnes et al [10] and Ajayi & Horn [15] observed a decrease in K_sat_ of sand mixed with wood BC by 92% and 23–82%, respectively. Jeffery et al [16] and Obia et al [17] on the other hand found no effect on sand mixed with BC from herbaceous feedstock and corn cob, respectively. Also, in loamy soils, Laird et al [18] and Ajayi & Horn [15] found no effect of wood BC at 2% dose on K_sat_ whereas at high dose of 10%, Ajayi & Horn [15] found an increase of 77%. These mixed results demonstrate that there is a need for further research, in particular for Vertisols, given their global importance for agricultural production e.g. in Indonesia, where this study was conducted, India [4,5,19] and in Africa [2].

A few studies exist showing that BC produced from different feedstocks increases the proportion of macro-pores, i.e., pores with diameter of >0.3 mm and holds water in the soil at a pressure <1 kPa [20], in repacked clayey soil [14] as well as in clay soil under field conditions [6]. An increase in the proportion of macro-pores is an indication of potential increase in soil aeration since such pores do not hold water against free drainage, provided the pores are interconnected. Indeed the air capacity, which is the air-filled volume of soil at field capacity (moisture content of the soil at 10 kPa pressure) has been reported to increase upon application of BC produced from fruit tree pruning to clayey soil [14]. However, results for kaolinitic clayey soil showed a quadratic pattern of macro-pores upon application of wood BC at a rate of up to 32 t ha^-1^ in Brazil [21]. Here, the macro-porosity showed a decrease at lower rates of BC addition, reaching a minimum at 16 t ha^-1^, and then increasing at higher rates.

Alongside drainage and soil aeration, available water capacity, which is the difference between moisture at field capacity and permanent wilting point, is a soil property that is important for agricultural production. Recent literature showed mixed results of the effect of BC amendment on available water capacity, moisture at field capacity and permanent wilting point of clayey soils [6,11,14,22]. Lu et al [22] observed an increase in available water capacity and moisture at field capacity and wilting point upon addition of rice husk BC to a Vertisol. Sun & Lu [6] found an increase in available water capacity upon addition of straw BC to a clayey soil, whereas the addition of wastewater sludge and woodchips BC had no effect. Castellini et al [14] on the other hand found a 10% increase in available water upon addition of 1% BC while a decrease of about 15% was observed upon addition of 3% BC. This shows the complexity of the way in which BC can affect these soil properties.

The possible alleviation of poor drainage and increased aeration of Vertisols following BC application may increase productivity of the soil. Limited data are available on the effect of BC amendment on crop yields grown in clayey soils. In a study involving modelling of yield of durum wheat, variable effect of BC was found, with an increase in yield only at low dose of BC [14]. Similarly, soybean grain yield increased, but only for the first few years after BC application in weathered clayey soil [23]. On the other hand, Carvalho et al [21] observed a decrease in rice yields with increasing wood BC rates in the absence of nitrogen fertilization in a clayey Ferralsol in Brazil. Asai et al [13] also observed a decrease in rice yield at 16 t ha^-1^ dose in clayey soil in Laos. Given the variation in the yield data, there is a need for further assessment of crop yield response to BC application in clayey soils.

We hypothesize that BC addition increases aeration and improves drainage of Indonesian Vertisols. In addition, BC is hypothesized to increase available water capacity by altering soil pore size distribution. Increased soil aeration and improved bulk density are expected to increase maize yields. To test these hypotheses, a field experiment was set up in Indonesia to test the effect of BC on maize yields. Soil samples from the same site were taken and mixed with various dosages of BC in the laboratory and exposed to two wetting and drying cycles, whereupon air capacity, K_sat_ and water retention were measured.

## Materials and methods

### Biochar

Two BCs, one made from *Gliricidia sepium* and the other from *Eupatorium adenophorum* were used in this study. Gliricidia BC was used in the field experiment in Indonesia while a similar BC made from Eupatorium was used for the laboratory experiment. Gliricidia is a common edge crop in the area where the field experiment was conducted and its pruning could be used as BC feedstock while Eupatorium was considered the optimal feedstock for BC in Nepal due to its abundance and its threat to forests [24]. Eupatorium BC was used in the laboratory experiment because the Gliricidia BC was fully utilized in the field. In addition, Eupatorium has a similar woody origin and similar physical properties and was hence chosen (Table 1). Gliricidia twigs were pyrolyzed at a temperature of 350 °C for 24 hours in West Timor, Indonesia in a simple kiln described in earlier studies [25,26]. Eupatorium stems 1–2 cm thick were pyrolyzed in a flame curtain kiln in the ground. The peak temperature was about 550 °C with overall pyrolysis time of about 2 hours. More details about the Eupatorium BC were reported earlier by Pandit et al [26] and Cornelissen et al [27]. The properties of the two BC are presented in Table 1.

**Table 1:** Properties of heavy clay soil and biochars.

Soil/Biochar	Soil	Gliricidia BC	Eupatorium BC^1^
TOC (%)	3.8	74.1	71.4
Total N (%)	0.32	0.66	0.66
Total H (%)	1.37	2.44	2.16
C/N	12.2	112.3	108.2
K^+^ (cmol_c_ Kg^-1^)	0.61	-	92.1
Ca^2+^ (cmol_c_ Kg^-1^)	56.5	-	18.7
Mg^2+^ (cmol_c_ Kg^-1^)	5.93	-	8.4
Na^+^ (cmol_c_ Kg^-1^)	0.08	-	12.6
CEC (cmol_c_ Kg^-1^)	63	7.1	127
pH H_2_O	7.3	9.4	10.4
pH CaCl_2_	6.9	8.9	9.2
Sand	6.0	-	-
Silt	22.7	-	-
Clay	71.4	-	-

^1^ data was obtained from Cornelissen et al. (2016) and Pandit et al. (2017). The soil is from plough layer of heavy clay in West Timor, Indonesia.

### Field experiment

The field experiment was conducted on a private land with permission of the owner and did not involve endangered or protected species. The site was established in Oebola Village, Fatuleu District, West Timor, Indonesia (S10° 03' 34'' E124° 00' 03'') in February 2012. The soil at the experimental site is a heavy clay classified as a Vertisol and is therefore characterized by swelling and shrinking upon wetting and drying, respectively. The experimental treatments included different BC rates and two BC application methods; planting basins with length*width*depth of 40*15*20 cm, respectively at a spacing of 90*70 cm or 20 cm deep rip line with inter row spacing of 90 cm. The BC was mixed with soil in the basins or rip lines at rates of 0 t ha^-1^, 5 t ha^-1^, 10 t ha^-1^. The 5 and 10 t ha^-1^ correspond to 2.5 and 5% BC rates respectively based on the fact that the BCs were concentrated in planting basins and rip lines. Each of the BC rates for the two application methods was replicated three times in a randomized block trial design. The soil was cropped with maize for two consecutive seasons of February—April 2012 and February—April 2013 without re-applying the BC. The harvest of the experiment for both seasons was carried out between 28 April and 1 May.

### Preparation of soil and biochar for laboratory experiment

Top soil from the plough layer was collected from the same experimental field in West Timor, Indonesia. The soil was air-dried at room temperature for ten days by spreading on trays in the laboratory. The gravimetric moisture content of the air-dry soil was determined to be 8.3% by oven drying at 105 °C. The air-dry soil as well as the Eupatorium BC was crushed and passed through a 2 mm sieve. The BC was then uniformly mixed with soil at a rate of 2.5%, 5% and 10% dry weight basis after correcting for soil moisture contents. A control without the addition of biochar was also prepared and treated in the same way. The soil samples without BC and those amended with BC were separately placed in metallic containers of about 30 cm length * 15 cm width * 10 cm depth without any compaction. The soil in the metallic containers were incubated for 14 days during which it underwent two wet-dry cycles in the laboratory at room temperature. Wetting was done by spraying water on the soil surface until water appeared on the surface showing saturation. Free drainage of excess water from the container occurred through the perforated bottom of the container. The soil and soil-BC mixtures were then allowed to dry until cracks started appearing on the soil surface, which took seven days. Re-wetting was carried out after these seven days. The incubations mimic "undisturbed soil" conditions, as would occur in the field after BC incorporation into the soil. Two metallic containers with "undisturbed soil" for each treatment (control, 2.5%, 5% and 10% BC) were prepared where core ring samples (three for water retention and four for K_sat_) were taken. Core ring samples of this "undisturbed soil" were taken on the 14^th^ day from all treatments. By the time of core ring sampling, the gravimetric moisture contents were 43.8%, 49.0%, 49.9% and 50.0% for control, 2.5%, 5% and 10% BC treatments, respectively. The samples were then used for bulk density, K_sat_, water retention and air capacity measurements.

### Measurement of K_sat_

The K_sat_ of the soil was measured using a laboratory permeameter using a constant water head (Eijkelkamp Agrisearch Equipment, the Netherlands). Four 100 cm^3^–core ring samples were taken from each of the containers under different treatments. K_sat_ (cm day^-1^) was calculated from Darcy's law, Eq 1.

$$K_{sat}=\left(V*L\right)/\left(A*t*h\right)$$ (1)

where *V* is the volume of water (cm^3^) collected for a duration of time *t* (days) flowing through the core sample of length *L* (cm) and cross sectional area *A* (cm^2^), *h* (cm) is the water level difference inside and outside core ring holder (also called hydraulic head). The measured K_sat_ was then used as the potential drainage capacity of the soil.

### Measurement of soil water retention capacity

The water retention capacity was measured using a sand box (Eijkelkamp Agrisearch Equipment, the Netherlands) and pressure plate apparatus (Soil moisture Equipment, Santa Barbara, CA). Three 100 cm^3^ core ring soil samples were taken from each of the treatment incubated in the laboratory. The core ring soil samples were saturated with water overnight and placed in the sand box. The saturated samples in the sand box were then drained by successively applying suction pressures at 10 hPa, 30 hPa and 50 hPa. At each suction pressure, the weight of the sample was taken after equilibration, which took between 4–5 days, when no more water was coming from the samples. For the pressures of 100 hPa, 1000 hPa and 15000 hPa, samples were transferred to the pressure plate. Positive pressures were applied to the samples and weights taken after equilibration (5–7 days). The pycnometer was used to measure the air volume in the soil after sample equilibration at 100 hPa by using the relation between air pressure and volume (Boyle's law). The dry weight of the soil samples was determined by oven drying at 105 °C for 48 hours after recording equilibration weight at 1000 hPa pressure, in order to allow calculation of dry bulk density and water contents at this and the smaller pressures. The oven-dried samples were then crushed and passed through a 2 mm sieve. Small plastic cylinders were filled with the sieved samples, placed on a pressure plate in the pressure chamber and saturated before applying a pressure of 15000 hPa. Weights were taken after equilibration and after oven drying to determine water content at this pressure.

In this study, the field capacity and wilting point was measured at pressures of 100 and 15000 hPa, respectively while available water capacity was the difference between the two. The air capacity, which is the air content of the soil at field capacity was measured at 100 hPa pressure using a pycnometer. The total porosity was calculated as the sum of field capacity and air capacity of the soil.

The water retention data was fitted using the van Genuchten [28] model (Eq 2) from which pore size distribution was derived using capillary equation (Eq 3) as earlier described by Obia et al [9].

$$\theta =\theta_{r}+(\theta_{s}-\theta_{r}){\left[1+{\left(\alpha \left|h\right|\right)}^{n}\right]}^{-m}$$ (2)

where, *θ* is the water content (cm^3^ cm^-3^) at a given pressure *h* (hPa) while *θ*_*r*_ and *θ*_*s*_ are the residual and saturated water content respectively. Residual water content was set at zero during modelling. *α*, *n* and *m* are van Genuchten parameters.

$$r=\frac{2\gamma .\text{cos}\theta }{\rho gh}*10^{8}$$ (3)

where, *r* is pore radius (μm), *h* is pressure in hPa, *γ* is water surface tension (0.0728 N m^-1^ at 20 °C), *ρ* is water density (1000 Kg m^-3^), *g* is gravitational constant (9.81 m s^-2^) and *θ* is contact angle between water and solid ~ 0.

### Statistical analysis

The data was analyzed with R statistical software [29] whereas graphs were drawn using SigmaPlot software 10 (Systat Software, Point Richmond, CA, USA). For the data interpretation of the four dosage experiment in the laboratory study, we preferred a dose response model (regression model) over a simple ANOVA, since it provided an indication of the effect of biochar at all dosages between 0% and 10%, not only the ones actually chosen in the experiment. Bulk density, total porosity, macro-porosity, moisture at field capacity and permanent wilting point and available water capacity were therefore analyzed by fitting a linear regression model, in which the BC dose was treated as a continuous explanatory variable. Air capacity and K_sat_, were analyzed by fitting a quadratic model using BC dose as the continuous explanatory variable. The BC dose at which the minimum air capacity and K_sat_ was observed was calculated from–*b/2a*, where *a* and *b* were the linear and quadratic coefficient, respectively. Biomass and grain yield data were analyzed using ANOVA with both BC application method and BC doses as categorical explanatory variables. The BC application method was not a significant factor and therefore it was removed from the model resulting in an increase in the number of replicates from three to six for each BC rate. Mean values were separated using Tukey's test at 5% level of significance.

## Results

### Bulk density of the heavy clay soil

**Fig 1:**
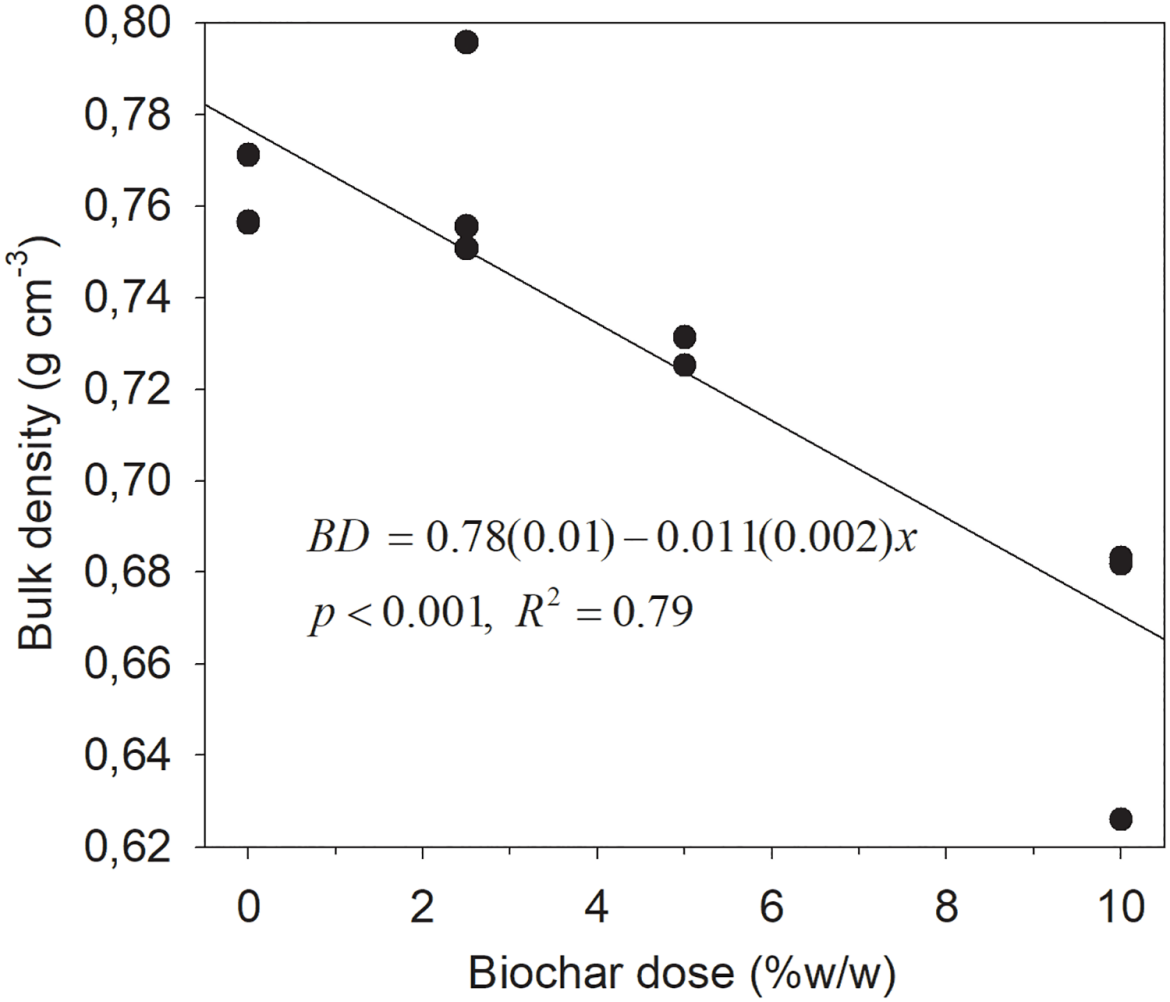
Bulk density of the soil as function of BC dose, n = 3. The soil is from the plough layer of a heavy clay in West Timor, Indonesia.

The bulk density of the soil prepared in the laboratory was 0.78±0.01 g cm^-3^ and decreased linearly with increasing BC dose by 0.011±0.002g cm^-3^ (p = 0.0001; R^2^ = 0.79) per percent BC added (Fig 1). Using field samples with known volume showed that this soil had a bulk density of only 0.66 g cm^-3^.

### K_sat_ of the heavy clay

**Fig 2:**
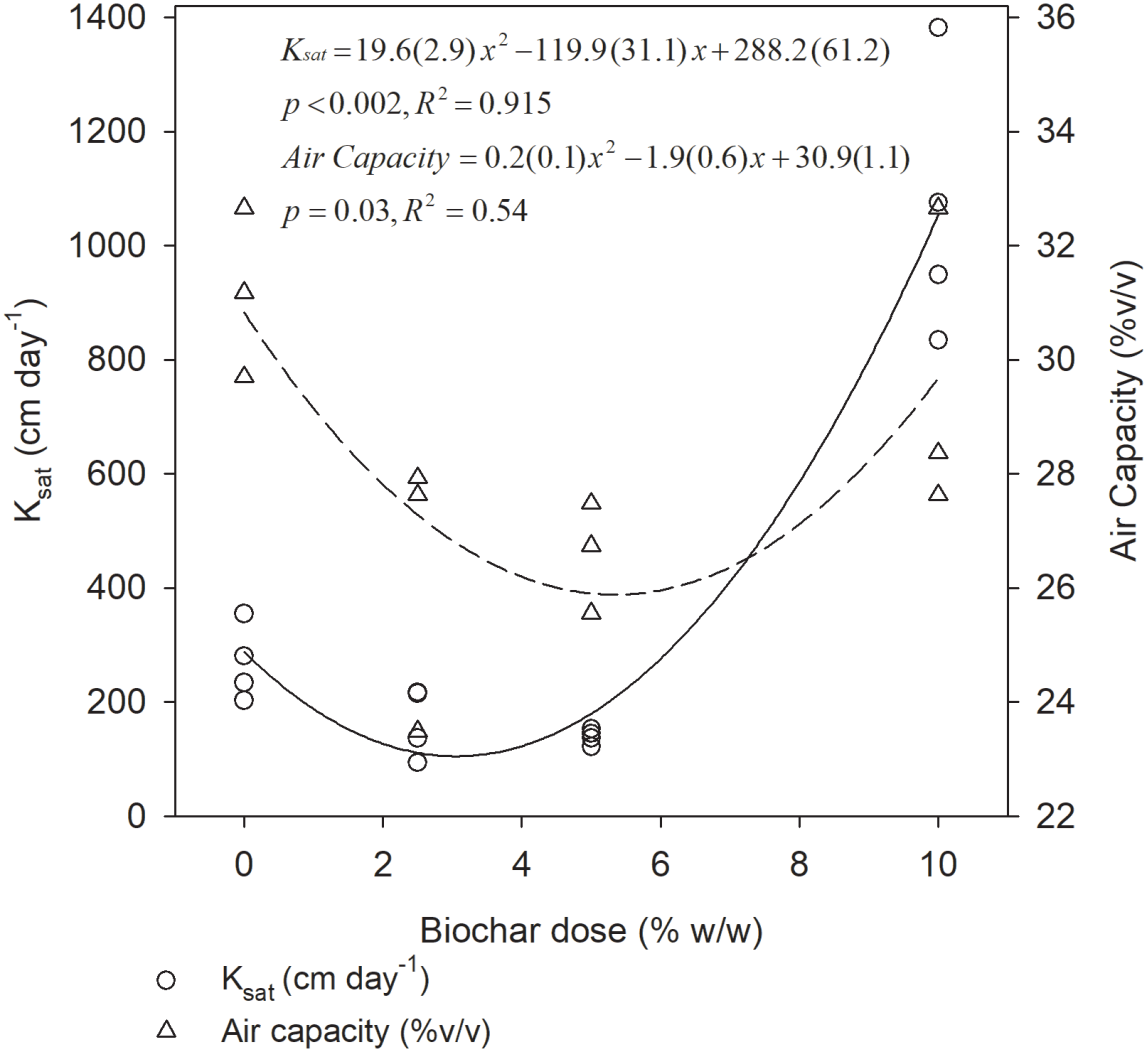
K_sat_ and air capacity of heavy clay soil at various BC doses, for four and three replicates, respectively. The numbers in brackets in the equations are the standard errors. The soil is from plough layer of heavy clay in West Timor, Indonesia.

Biochar significantly (p<0.05) reduced the K_sat_ of the soil at low doses while at high doses, an increase was observed (Fig 2). The nonlinear decrease in K_sat_ at low doses and increase at high doses was well described by a quadratic function (R^2^ = 0.915). K_sat_ was reduced from 288±61 cm day^-1^ in control soil to a minimum of 105 cm day^-1^ at 3.1% BC addition. This decrease in K_sat_ was by 100 cm day^-1^ between 0 and 1% BC with the slope becoming subsequently less steep (by 2*19.6±2.9 cm day^-1^ –quadratic coefficient) for each additional dose of BC until the minimum of 105 cm day^-1^. Above minimum K_sat_, the slope increased at larger rate with increased BC dose.

### Air capacity and macro-porosity

**Fig 3:**
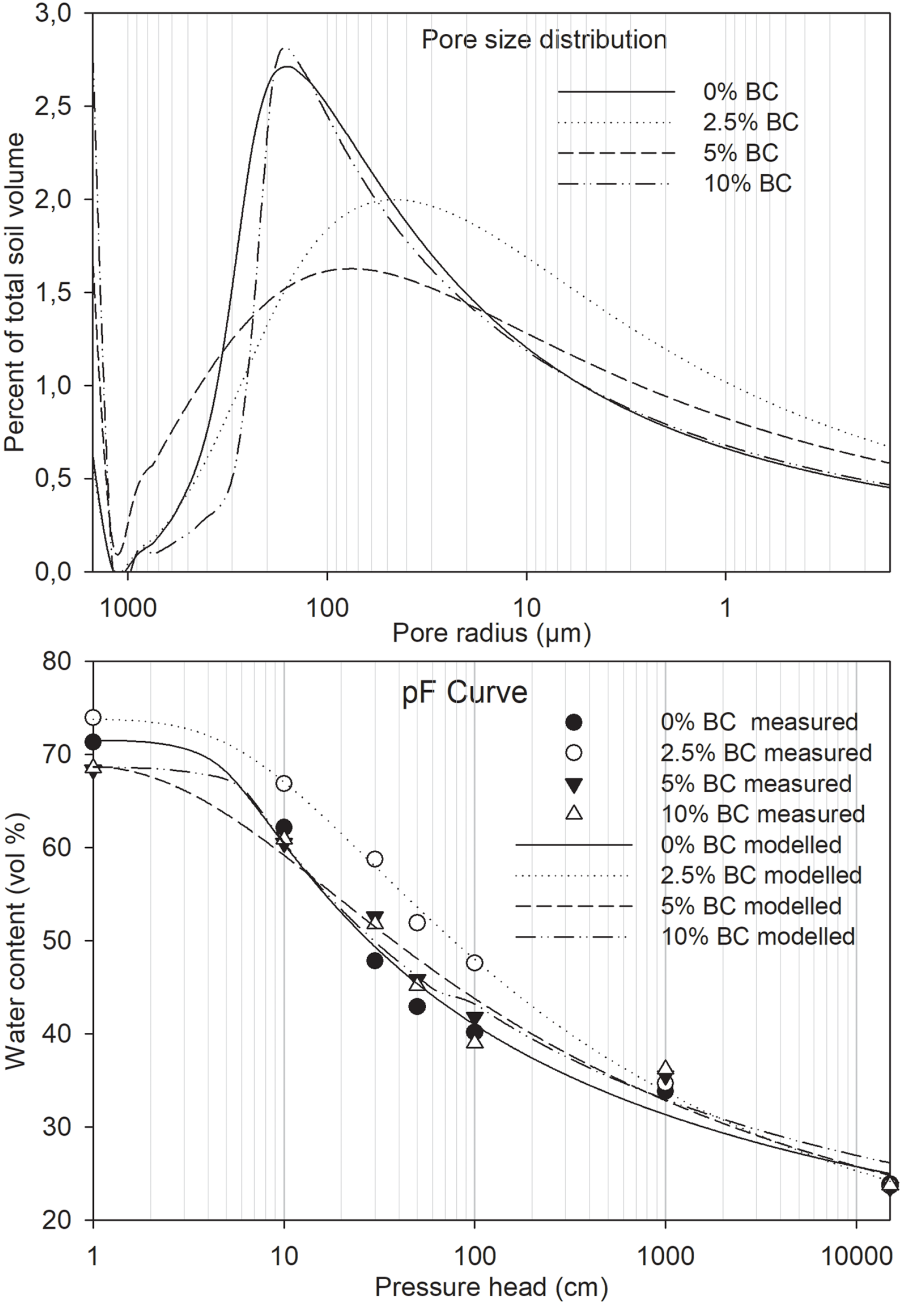
Water characteristic curves (pF) and pore size distribution of heavy clay soil amended with Eupatorium BC. The pore radius correspond directly to the pressure head in the pF curve. The distribution of pores <0.1 μm, which constituted ~25% of the total soil volume is not shown because water held by these pores is not available to plants. The soil is from plough layer of heavy clay in West Timor, Indonesia.

Air capacity, the volume of soil pores filled with air at field capacity, was affected by BC application. Similar to the effect of BC on K_sat_, the air capacity of the soil was best described by a quadratic function (p = 0.03, R^2^ = 0.54 compared to p>0.05 and R^2^ of 0.01 for a linear fit). The air capacity of the soil was 30.8±1.1% v/v and decreased with increasing BC dose at low doses while increasing at higher BC doses (Fig 2). The decrease was by 1.7% v/v between zero and 1% BC and subsequently becoming smaller (by 2*0.175% v/v—quadratic coefficient) per percent BC from 1% BC until a minimum of 25.9% v/v at 5.3% BC. The changes in air capacity were also reflected in the shift in pore size distribution (Fig 3). Pores with radius of 100–300 μm had the highest density in control soil and there was a strong shift of this size to pores of 0.1–100 μm radius upon addition of 2.5% BC meanwhile the shift in pore size distribution was on both sides of 100–300 μm pore-size window upon addition of 5% BC. The 10% BC did not change the pore distribution significantly relative to the control soil, except for pores of ≥ 3mm diameter, which became 2% more abundant. The macro-porosity of the soil was 8.9±0.6% (v/v) and was not affected by BC, p = 0.20 (data not shown).

### Field capacity, permanent wilting point and available water capacity

**Fig 4:**
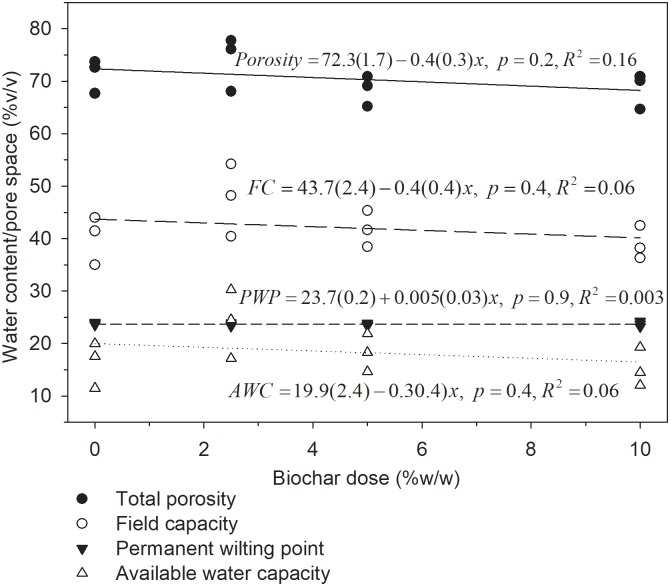
Total porosity, field capacity, permanent wilting point and available water capacity as a function of BC dose, n = 3. The soil is from plough layer of heavy clay in West Timor, Indonesia.

The soil porosity was high at 72.3% and was not affected by BC amendment, p = 0.2 (Fig 4). Likewise, water retention properties important for upland farming, such as moisture at field capacity and permanent wilting point and available water capacity were 43.7%, 23.7% and 19.9%, respectively, and were not affected by BC, p>0.05 (Fig 4).

### Maize yields for 2012 and 2013 seasons

**Fig 5:**
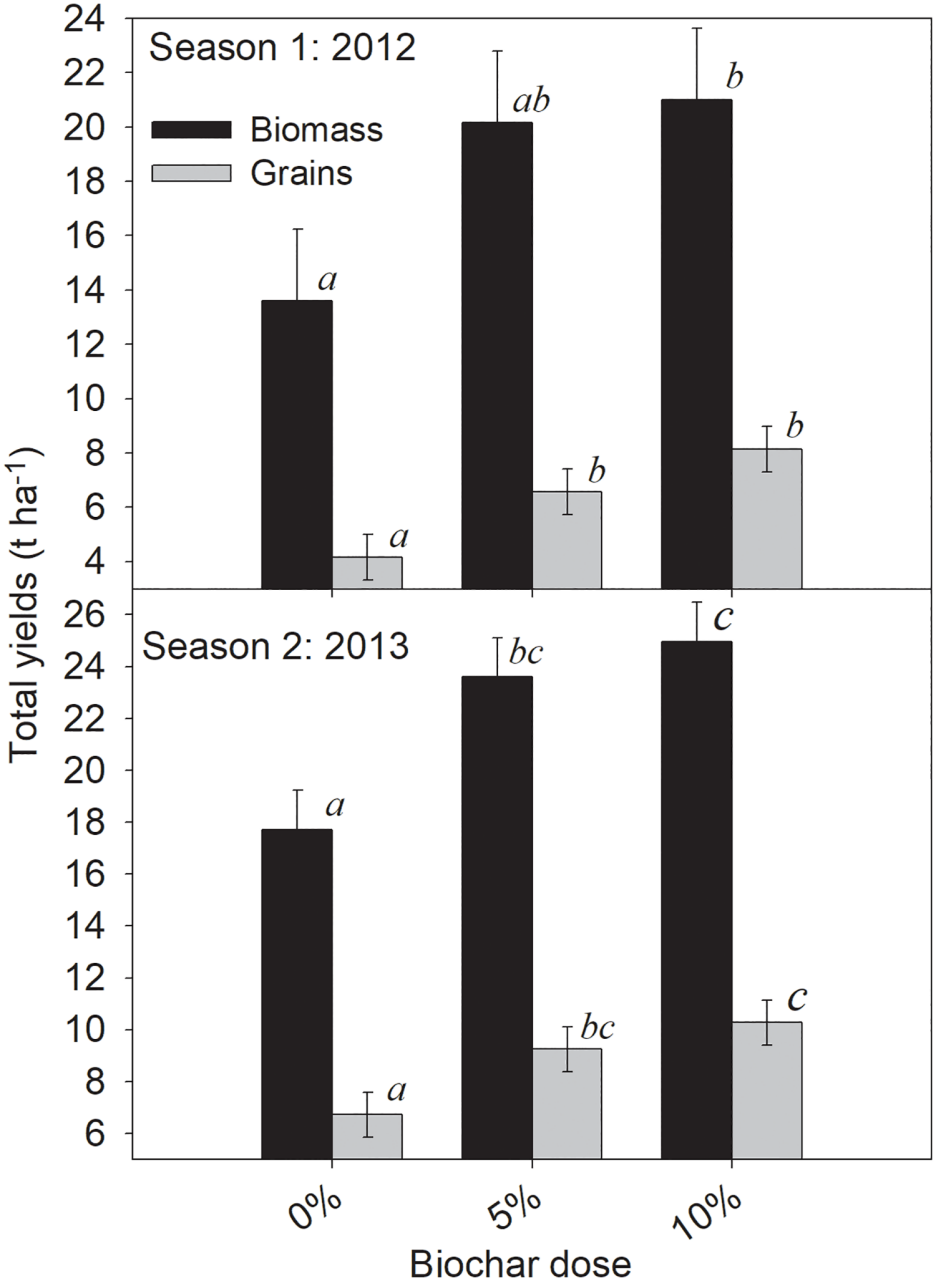
Maize yields from field plots amended with BC. 5 and 10 t ha^-1^ BC, corresponding to 2.5 and 5% BC, respectively. Means followed by the same letter are not significantly different, Error bars are the standard error, n = 6. Experiment was conducted in heavy clay in West Timor, Indonesia.

The mean biomass yields for biochar-amended plots were significantly larger than those for the control plots (p<0.05) except for BC at 2.5% in the first year after amendment (2012; Fig 5). There was greater biomass yield in 2013 (18 to 25 t ha^-1^) compared to 2012 (14 to 21 t ha^-1^). For the grains, the mean yields were significantly greater for all the BC amended plots compared to the control in both 2012 and 2013. Similar to the pattern of biomass yield, the grain yield were also larger in 2013 (7 to 10 t ha^-1^) than in 2012 (4 to 8 t ha^-1^).

## Discussion

Biochar amendment increased both biomass and grain yield of maize for two consecutive years after a one time application in this study. The yield increase over two years implies that BC has positive effects on this heavy clay soil for more than one cropping season. The increase in yield could be due to the decrease in soil bulk density with increased BC dose (Fig 1) which could result in an improvement in root growth, as has been shown for some soils [30]. This observed yield increase is consistent with earlier reports for clayey soils for other crops e.g. durum wheat [14] and soybeans during the first few years after BC application [23]. The drainage potential (K_sat_) and air capacity, the two physical properties of major importance in heavy clays (wet system in rainy season), decreased to a small extent under laboratory condition within the range of BC doses (0–5%) used in the field (Fig 2). However, at high BC dose, both K_sat_ and air capacity increased coupled with changes in pore size distribution, which may favor yield increases under field conditions. Therefore, the maize response is in accordance with the hypothesis that BC increases its yield although it was not fully explainable by changes in physical properties of the soil.

The K_sat_ of the studied soil was 288 cm day^-1^, in the range of 43–432 cm day^-1^ which is believed to be optimal for non-degraded fine-textured agricultural soils [20,31]. The macro-porosity, which contributes most to water-conducting pores during drainage, was also within the proposed optimum of 5–10% for non-compacted fine-textured soils for maximum crop production [20,32]. Likewise, air capacity, which includes the macro-porosity of 8.9%, was 30.8%, and thus above the proposed minimum of 15% [32] for proper crop growth. The total porosity of the studied soil was large (72%), hence the low bulk density of 0.78 g cm^-3^ (Fig 1), which is below optimal range of 0.9–1.2 g cm^-3^ for fine-textured soils [20,32,33]. The bulk density and porosity was on the low and high end, respectively, of what is expected of heavy clay, which may be attributed to high moisture contents of 43% for control soil and ~50% for BC amended soil (≤100 hPa or cm pressure, Fig 3) at the time of core ring sampling [2]. This means the soil was sampled at near full swelling state hence low dry bulk density and high porosity. The available water capacity of 19.9% was in the lower range of what is considered ideal of ≥ 20% [20] (Figs 2, 3 and 4).

Soil bulk density of <0.9 g cm^-3^ is believed to compromise soil-root contact and plant anchoring as discussed in Reynolds et al [31]. However, the low bulk density here may not affect plant anchoring due to the stickiness of the heavy clay that minimize root slippage from soil. In fact, Easson et al [34] reported a higher tensile force (4.8 N) required to pull roots from finer-textured sandy clay loam than from coarser sandy loam (3.9 N), suggesting stronger root anchorage in finer soils. We observed loss of stickiness (not quantitatively determined) at doses ≥ 5% BC, which are not very relevant for field application. Similar changes in mechanical properties of Vertisols related to stickiness have also been reported before [22,35].

The bulk density decreased linearly with increasing dose of BC, as total porosity remained unaffected likely due to already high porosity of the soil (Figs 3 and 4). This is not in agreement with the known negative correlation between bulk density and porosity. The BC had a lower bulk density (0.35 g cm^-3^) than the soil (0.78 g cm^-3^), indicating that weight dilution could have contributed to the decrease of soil bulk density. In fact, weight dilution nearly completely (~90%) accounted for the observed decrease in bulk density, in contrast to earlier study on light-textured soil [9], where soil aggregation contributed to a decreasing bulk density by an increase in porosity.

Since total porosity was not affected by BC amendment, the quadratic pattern of K_sat_ and air capacity (Fig 2) resulted from a shift in pore size distribution towards smaller pores at low doses (up to 5%, as in the field experiment) and towards larger pores at higher doses (Fig 3). The decrease in K_sat_ and air capacity at low doses of BC and increase at high doses (quadratic pattern, Fig 2) in this study does indicate that different mechanisms were dominating the effect of BC between low and high doses. At low doses of BC, it is possible that BC particles were mainly filling the inter-aggregate spaces in the soil increasing the proportion of smaller pores (Fig 3). In contrast, at doses > 5% BC, the large amounts of BC in the soil-BC mixture may have introduced large inter-particle pores of BC that contributed to increasing K_sat_ and air capacity. This is possible given that coarser BC passed through a 2 mm sieve was mixed with a very fine-textured soil with 71.4% clay and only 6% sand. The higher K_sat_ at 5% and 10% BC could be related to the increase in macro-pores of ≥ 3 mm diameter by 1% and 2%, respectively (Fig 3) due to coarser BC. Such large pores are very important for water transport in soils. Similar quadratic responses of soil physical properties to woody BC application in clayey soils [21,23] and coarse-textured soil [36] have been reported earlier. The quadratic relations in these studies were found only from the start of the experiment to the second year after experimental setup, similar to our freshly amended soil. Macro-porosity decreased at low doses for clayey soils but the minimum values were attained at a much lower dose of BC of <1% compared to our study at 3–5% BC (Fig 2). The macro-porosity in our study appeared to follow the same quadratic pattern reflected in the air capacity and K_sat_. Therefore, the hypothesis that BC would increase K_sat_ and aeration of Indonesian Vertisols was confirmed only for high doses of BC.

Biochar did not affect moisture at field capacity and permanent wilting point and available water capacity (Fig 4). Similar findings have also been reported for clayey soils e.g. Sun and Lu [6] who found no effect of wastewater sludge and woodchip BC on available water capacity in a Vertisol. Therefore, within the limits of a laboratory experiment, the hypothesis that BC would increase available water capacity by altering pore size distribution of our Vertisol was falsified.

## Conclusions

The heavy clay soil investigated here had high porosity and low dry bulk density. The porosity was not affected by BC amendment, but the bulk density of the soil decreased with increasing BC doses, primarily due to weight dilution caused by the BC. The K_sat_ of the soil was within what is considered optimal for fine-textured soils. Air capacity of the soil was also high. Both K_sat_ and air capacity followed a quadratic pattern with increasing BC doses. K_sat_ and air capacity decreased, reaching minimum values at 3–5% BC application, and increased at higher doses due to alteration in pore size distribution. Moisture at field capacity and permanent wilting point and available water capacity were not affected by BC. In the field, BC amended plots had higher maize yields compared to the control partly due to improved bulk density. There was no significant difference between 2.5 and 5% BC doses, which implies that a BC dose of more than 2.5% in the field in this case does not bring an additional gain. The 2.5% BC amendment only minimally reduced K_sat_ and air capacity and hence, it does not affect the productivity of heavy clay negatively.
